# Association between prediabetes and thyroid cancer risk: A meta-analysis

**DOI:** 10.17305/bb.2025.12744

**Published:** 2025-09-02

**Authors:** Yi Shen, Xiaoen Li, Yupan Chen, Xujie Han, Rongli Xie

**Affiliations:** 1 Department of General Surgery, Ruijin Hospital Lu Wan Branch, Shanghai Jiaotong University School of Medicine, Shanghai, China

**Keywords:** Prediabetes, hyperglycemia, thyroid cancer, incidence, meta-analysis

## Abstract

Prediabetes, characterized by intermediate hyperglycemia, is increasingly prevalent worldwide. While diabetes has been associated with a heightened risk of various cancers, the relationship between prediabetes and thyroid cancer remains ambiguous. This meta-analysis sought to assess whether prediabetes correlates with an elevated incidence of thyroid cancer. A systematic literature search was conducted across PubMed, Embase, Web of Science, Wanfang, and CNKI to identify longitudinal studies that compared the incidence of thyroid cancer in individuals with prediabetes to those with normoglycemia. Risk ratios (RRs) with 95% confidence intervals (CIs) were aggregated using a random-effects model. Subgroup and sensitivity analyses were performed to identify potential effect modifiers. Six prospective cohort studies, encompassing 5,743,849 participants, were included in the analysis. Overall, prediabetes was not significantly correlated with thyroid cancer incidence (RR = 1.04; 95% CI: 0.98–1.11; *P* ═ 0.23; *I*^2^ ═ 53%). Subgroup analyses revealed no significant variations based on age, sex, region, follow-up duration, or definition of prediabetes. Notably, a significant association was identified in studies utilizing cancer registries or validated clinical diagnoses (RR = 1.29; 95% CI: 1.04–1.60), in contrast to studies relying solely on ICD-10 codes (RR = 1.01; 95% CI: 0.98–1.05; *P* for subgroup difference = 0.03). In conclusion, prediabetes was not linked to a significantly increased risk of thyroid cancer overall. However, a potential association was noted in studies employing clinically validated cancer diagnoses. These findings, derived from observational cohorts, should be interpreted cautiously, and further prospective research is necessary to elucidate any causal relationship.

## Introduction

Thyroid cancer is the most prevalent malignancy of the endocrine system, exhibiting a consistent rise in global incidence over recent decades [1–3]. It is estimated that thyroid cancer constitutes approximately 3% of all new cancer diagnoses worldwide, with a higher prevalence observed among women and in high-income countries [4, 5]. Although most differentiated thyroid cancers yield a favorable prognosis, with a 5-year survival rate exceeding 90%, the risk of recurrence and disease progression remains significant in certain subgroups, particularly those with aggressive histological subtypes or distant metastases [6, 7]. Treatment primarily involves surgical resection, often followed by radioactive iodine therapy and thyroid hormone suppression [8]. Nonetheless, despite advancements in management, challenges persist in accurately predicting which individuals are at an elevated risk of developing thyroid cancer [9, 10]. Identifying modifiable risk factors and at-risk populations is essential for effective early detection and primary prevention strategies.

Metabolic disturbances, including hyperglycemia, insulin resistance, and chronic low-grade inflammation, are increasingly recognized as significant contributors to carcinogenesis [11, 12]. Recent evidence indicates that glucose metabolism may also play a role in thyroid tumorigenesis [13, 14]. Mechanistically, insulin resistance and elevated insulin-like growth factor levels can stimulate thyroid cell proliferation and inhibit apoptosis, potentially facilitating malignant transformation [15, 16]. Prediabetes, characterized by intermediate hyperglycemia that does not meet the diagnostic criteria for diabetes, is typically identified through impaired fasting glucose (IFG), impaired glucose tolerance (IGT), or elevated glycated hemoglobin (HbA1c) levels (generally between 5.7% and 6.4%) [17, 18]. With a global prevalence estimated to affect over 7%–10% of adults, prediabetes represents a critical stage for intervention to prevent not only the progression to diabetes but also potentially associated comorbidities, including cancer [19].

Numerous observational studies have examined the association between prediabetes and the incidence of various site-specific cancers [20]. Some studies indicate an increased risk for cancers of the colorectal [21], liver [22], pancreas [23], stomach [24], and lungs [25]. Additionally, accumulating evidence suggests a correlation between diabetes and a higher incidence of thyroid cancer [26–29]. However, the relationship between prediabetes and thyroid cancer remains ambiguous. Individual studies have produced inconsistent results, likely due to variations in study design, population characteristics, definitions of prediabetes, and methodologies for validating cancer outcomes [30–35]. To date, no comprehensive meta-analysis has quantitatively assessed whether individuals with prediabetes are at an elevated risk of developing thyroid cancer. Given the rising global prevalence of prediabetes and the increasing incidence of thyroid cancer, understanding this potential association is crucial for public health. Therefore, we conducted a systematic review and meta-analysis of longitudinal studies to evaluate the relationship between prediabetes and the incidence of thyroid cancer and to investigate the influence of study-level characteristics on this association.

## Materials and methods

This meta-analysis was conducted in accordance with the PRISMA 2020 statement [36, 37] and the Cochrane Handbook for Systematic Reviews [38]. These guidelines informed the development of the protocol, data collection, statistical synthesis, and reporting. The protocol has been prospectively registered in the PROSPERO database under the identifier CRD420251059664.

### Database search

To identify studies relevant to the objectives of this meta-analysis, we conducted a comprehensive search across PubMed, Embase, Web of Science, Wanfang, and the Chinese National Knowledge Infrastructure (CNKI) databases. Our search utilized an extensive set of terms, including: (1) “prediabetes” OR “pre-diabetes” OR “prediabetic” OR “pre-diabetic” OR “prediabetic state” OR “borderline diabetes” OR “impaired fasting glucose” OR “impaired glucose tolerance” OR “IFG” OR “IGT” OR “fasting glucose” OR “HbA1c”; (2) “thyroid”; and (3) “cancer” OR “neoplasm” OR “carcinoma” OR “malignancy” OR “tumor” OR “malignant”. The literature search was restricted to studies involving human participants and included only full-length, peer-reviewed articles published in English or Chinese. To ensure thorough coverage, we manually screened the reference lists of relevant original and review articles for additional eligible studies. The search encompassed the period from the inception of each database until April 12, 2025, with the complete search strategies outlined in Supplemental File 1.

### Study selection

The inclusion criteria were established based on the PICOS framework.

**Population (P):** Adults aged 18 years or older without a prior history of thyroid cancer.

**Exposure (I):** Participants diagnosed with prediabetes, defined by established criteria such as IFG, IGT, or mildly elevated HbA1c levels that fall below the diagnostic threshold for diabetes. Variations in prediabetes definitions across studies—including IFG (typically 100–125 mg/dL), IGT (2-hour glucose 140–199 mg/dL), and HbA1c (5.7%–6.4%)—were acknowledged and analyzed through subgroup analyses to assess the impact of diagnostic methods on the association with thyroid cancer risk.

**Comparison (C):** Participants with normoglycemia.

**Outcome (O):** Incidence of thyroid cancer during follow-up, compared between individuals with prediabetes and those with normoglycemia.

**Study design (S):** Longitudinal observational studies, encompassing cohort studies, nested case-control designs, and post-hoc analyses of clinical trials.

Exclusion criteria included reviews, editorials, meta-analyses, and studies involving children, those that did not evaluate prediabetes as an exposure, or those that did not report on thyroid cancer incidence. In instances of overlapping populations, the study with the most comprehensive dataset was selected.

### Study quality evaluation and data collection

The literature search, study selection, quality assessment, and data extraction were independently conducted by two reviewers, with any disagreements resolved through discussion with the corresponding author. Study quality was evaluated using the Newcastle–Ottawa Scale (NOS), which assesses three domains: participant selection, control for confounding, and outcome assessment [39]. The NOS assigns scores ranging from 1 to 9, with higher scores indicating superior quality; studies scoring 7 or above were classified as high quality. Extracted data encompassed study-level information (first author, publication year, country, and study design), participant characteristics (source of the population, sample size, mean age, and sex distribution), details on the diagnostic criteria for prediabetes, the number of participants with prediabetes at baseline, mean follow-up durations, the number of participants who developed thyroid cancer during follow-up, methods used to validate the diagnosis of thyroid cancer, and the covariates adjusted for in the association analyses.

**Figure 1.**
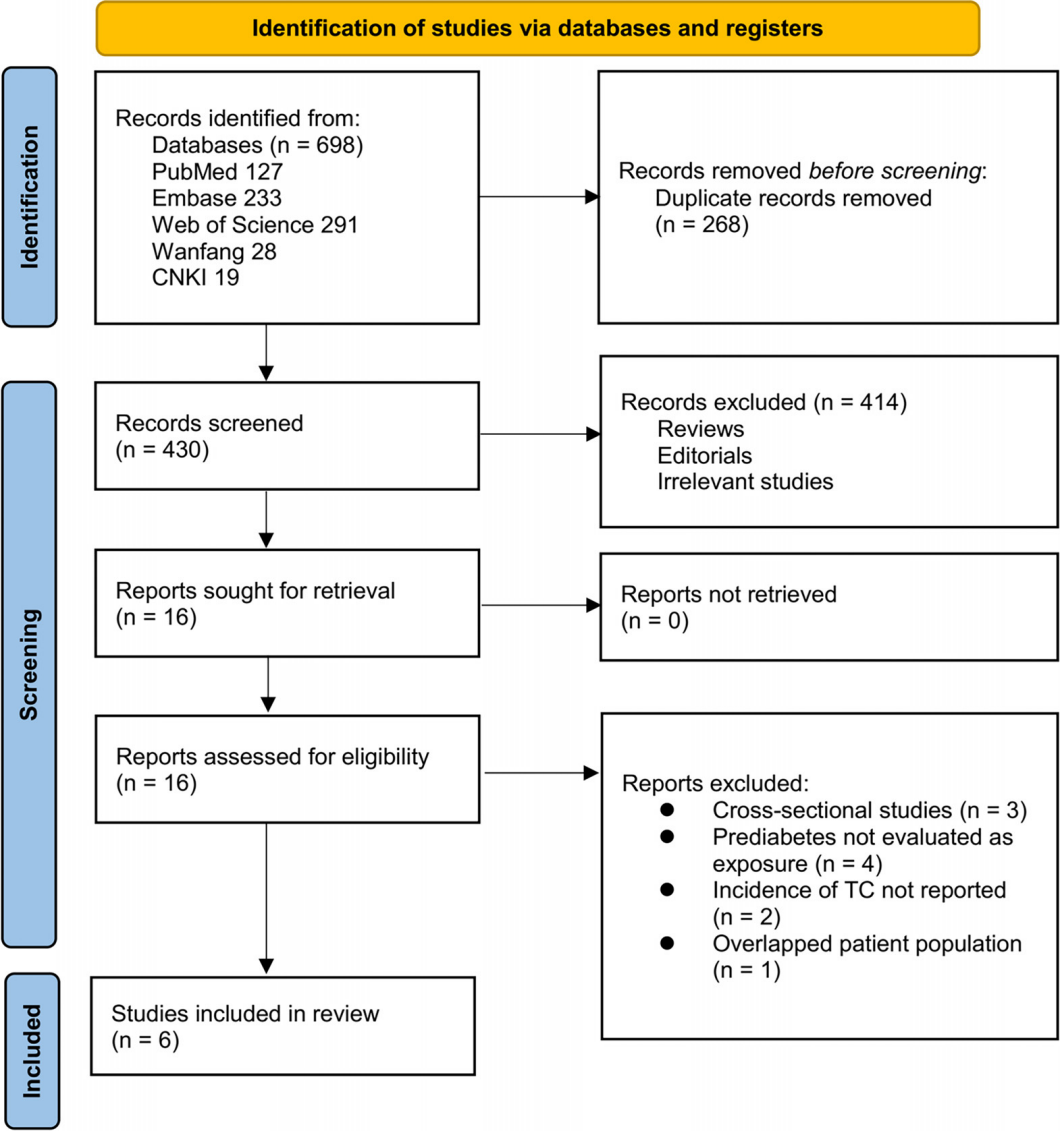
Flow diagram of study selection.

### Statistical analysis

The association between prediabetes and the incidence of thyroid cancer was assessed by pooling risk ratios (RRs) and their corresponding 95% confidence intervals (CIs), comparing individuals with prediabetes to those with normoglycemia. RRs and their standard errors were calculated from reported 95% CIs or *P* values when necessary, followed by log transformation to stabilize variance and normalize distribution [38]. Between-study heterogeneity was evaluated using the Cochrane *Q* test and the *I*^2^ statistic, with thresholds of <25%, 25%–75%, and >75% indicating low, moderate, and high heterogeneity, respectively [40]. A random-effects model was employed to account for variability across studies [38]. Specifically, the DerSimonian–Laird estimator with an inverse-variance (IV) weighting approach was utilized to pool the results. Sensitivity analysis was conducted by sequentially omitting each study to assess the stability of the pooled estimate. Subgroup analyses were performed to examine the impact of study-level characteristics, including study country (Asian vs Western), mean ages, sex, definitions of prediabetes, mean follow-up durations, and methods used to validate the diagnosis of thyroid cancer. Median values of continuous variables were employed to define subgroup cutoffs. Publication bias was assessed through visual inspection of funnel plots and formally tested using Egger’s regression test [41]. A *P* value < 0.05 was considered statistically significant. All statistical analyses were conducted using RevMan (version 5.1; Cochrane Collaboration, Oxford, UK) and Stata (version 12.0; Stata Corporation, College Station, TX, USA). The certainty of evidence for the primary outcome was evaluated using the GRADE (Grading of Recommendations, Assessment, Development, and Evaluation) framework, which assesses five domains: risk of bias, inconsistency, indirectness, imprecision, and publication bias [42]. Based on these criteria, the evidence was rated as high, moderate, low, or very low certainty.

## Results

### Study retrieval

The study selection process is depicted in Figure 1. A total of 698 potentially relevant records were initially identified through database searches and citation screening. After the removal of 268 duplicates, 430 records remained for title and abstract screening, resulting in the exclusion of 414 articles that did not align with the objectives of the meta-analysis. The full texts of the remaining 16 articles were independently assessed by two reviewers, leading to the exclusion of 10 studies for reasons detailed in Figure 1. Ultimately, six studies met the inclusion criteria and were incorporated into the quantitative synthesis [30–35].

**Table 1:** Characteristics of the included studies

**Study**	**Country**	**Design**	**Population characteristics**	**No. of participants**	**Mean age (years)**	**Women (%)**	**Diagnosis of PreD**	**No. of subjects with PreD**	**Mean follow-up (years)**	**No. of patients with TC**	**Methods for TC validation**	**Variables adjusted**
Rapp, 2006	Austria	PC	Adults >19 years from the general population	140,813	43	54.8	IFG	6754	8.4	70	Population-based cancer registry, histologically confirmed	Age, sex, smoking status, occupational group, BMI
Peila, 2020	UK	PC	Adults aged 40–69 years from the general population	476,517	56.2	54	Mildly elevated HbA1c (5.7–6.5%)	64,167	7.1	269	ICD-10 code	Age, sex, education, non-white race, smoking status, pack-years, alcohol intake, physical activity, BMI
Park, 2022	Korea	PC	Adults aged 40–70 years from the general population	4,658,473	51.5	47	IFG	1,570,425	6	47,325	ICD-10 code	Age, sex, and BMI
Nguyen, 2022	Korea	PC	Adults aged 40–79 years from general population	160,650	65.6	54.5	IFG	40,929	7.4	471	Self-reported physician-diagnosed TC validated by medical records/pathology reports	Age, sex, smoking, alcohol, physical activity, education; for women, also adjusted for menopausal status and hormone therapy
Miao, 2022	China	PC	Adults >20 years from the general population	259,657	67.2	65.3	IFG and/or IGT	31,568	4.4	78	Population-based cancer registry	Age and sex
Pasqual, 2023	USA	PC	Women aged 35–74 years with a sister who had breast cancer	47,739	55.4	100	Self-reported borderline diabetes (IFG, IGT, and/or mildly elevated HbA1c	NR^a^	12.5	249	Self-report confirmed by medical records/pathology reports	Age and BMI

Note: Summary of six prospective cohort studies included in this meta-analysis, detailing study design, populations, definitions of prediabetes, follow-up duration, thyroid cancer validation methods, and adjusted variables. a, Not reported in the original publication. This value was not required for meta-analysis, which pooled adjusted risk estimates and 95% CIs. Abbreviations: PC: Prospective cohort; PreD: Prediabetes; IFG: Impaired fasting glucose; IGT: Impaired glucose tolerance; HbA1c: Glycated hemoglobin; TC: Thyroid cancer; BMI: Body mass index; ICD-10: International Classification of Diseases, 10th Revision; NR: Not reported.

### Overview of the study characteristics

Table 1 summarizes the characteristics of the six studies included in this meta-analysis. A total of six studies [30–35], published between 2006 and 2023, were conducted in Austria, the United Kingdom, Korea, China, and the United States. All studies employed prospective cohort designs, with the majority involving adults from the general population [30–34], while one study focused specifically on women who had a sister with breast cancer [35]. The combined sample size consisted of 5,743,849 adults, with an average participant age ranging from 43 to 67.2 years; the percentage of female participants varied from 47% to 100%.

Prediabetes was defined in several ways: three studies [30, 33, 34] used IFG, one study [31] utilized mildly elevated HbA1c levels, and two studies [32, 35] employed combined criteria of IFG and/or IGT, or IFG, IGT, or mildly elevated HbA1c levels. The number of individuals with prediabetes ranged from approximately 6,754 to over 1.5 million, and follow-up durations varied from 4.4 to 12.5 years.

Thyroid cancer diagnoses were confirmed through population-based cancer registries [30, 32], self-reported clinically validated diagnoses [33, 35], or International Classification of Diseases (ICD) codes [31, 34], with two studies employing each of these methods. All studies reported multivariate-adjusted data, accounting for covariates such as age, sex, body mass index (BMI), smoking status, physical activity, and socioeconomic factors to varying extents. The methodological quality of the included studies, as assessed by the NOS, was generally high, with total scores ranging from 7 to 9 (Table 2). All studies received full points for representativeness, exposure ascertainment, and control for age and sex. However, variability was observed in the “Assessment of Outcome” domain: studies utilizing cancer registries or clinically validated diagnoses received full scores, while those relying on ICD-10 codes were downgraded due to potential outcome misclassification. This finding aligns with our subgroup analyses and highlights the importance of rigorous outcome validation in epidemiological research.

**Table 2:** Study quality evaluation via the Newcastle–Ottawa scale

**Study**	**Representativeness of the exposed cohort**	**Selection of the non-exposed cohort**	**Ascertainment of exposure**	**Outcome not present at baseline**	**Control for age and sex**	**Control for other confounding factors**	**Assessment of outcome**	**Enough long follow-up duration**	**Adequacy of follow-up of cohorts**	**Total**
Rapp, 2006	1	1	1	1	1	1	1	1	1	9
Peila, 2020	1	1	1	1	1	1	0	1	1	8
Park, 2022	1	1	1	1	1	1	0	1	1	8
Nguyen, 2022	1	1	1	1	1	1	0	1	1	8
Miao, 2022	1	1	1	1	1	0	1	0	1	7
Pasqual, 2023	1	1	0	1	1	1	0	1	1	7

Note: Overview of methodological quality assessment of the included studies using the Newcastle–Ottawa scale, covering domains of selection, comparability, and outcome.

### Association between prediabetes and the incidence of thyroid cancer

Four studies reported the association between prediabetes and the incidence of thyroid cancer in men and women separately [30, 32–34]. Consequently, these datasets were independently included in the meta-analysis, yielding a total of ten datasets. The strata were mutually exclusive and each provided risk estimates adjusted for relevant confounders. Statistically, treating independently adjusted, non-overlapping strata as separate units is a valid approach in meta-analysis and does not underestimate variance. Due to the absence of combined estimates across sexes in some studies, a sensitivity analysis based on pooled study-level estimates could not be conducted.

**Figure 2.**
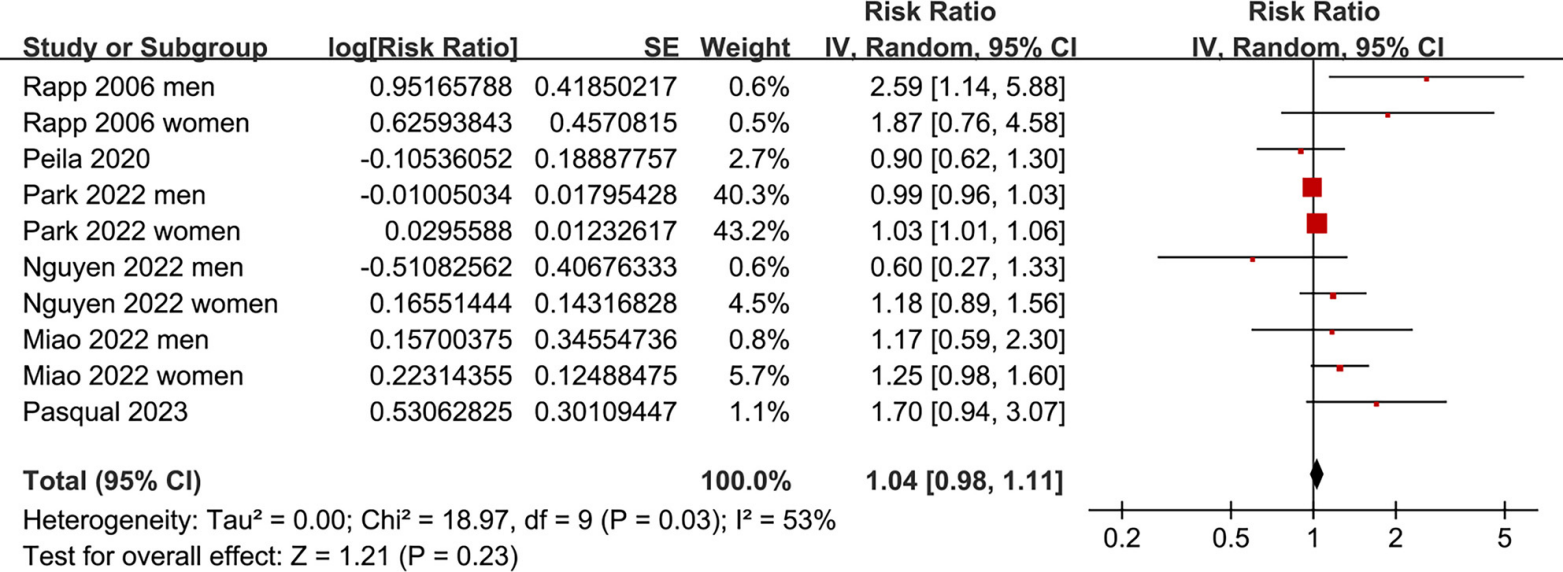
**Association between prediabetes and the incidence of thyroid cancer.** Forest plot showing RRs and 95% CIs for the association between prediabetes and thyroid cancer incidence across 10 datasets from six prospective cohort studies. Separate risk estimates were included for men and women where available. Abbreviations: RR: Risk ratio; CI: Confidence interval; SE: Standard error; IV: Inverse variance.

**Figure 3.**
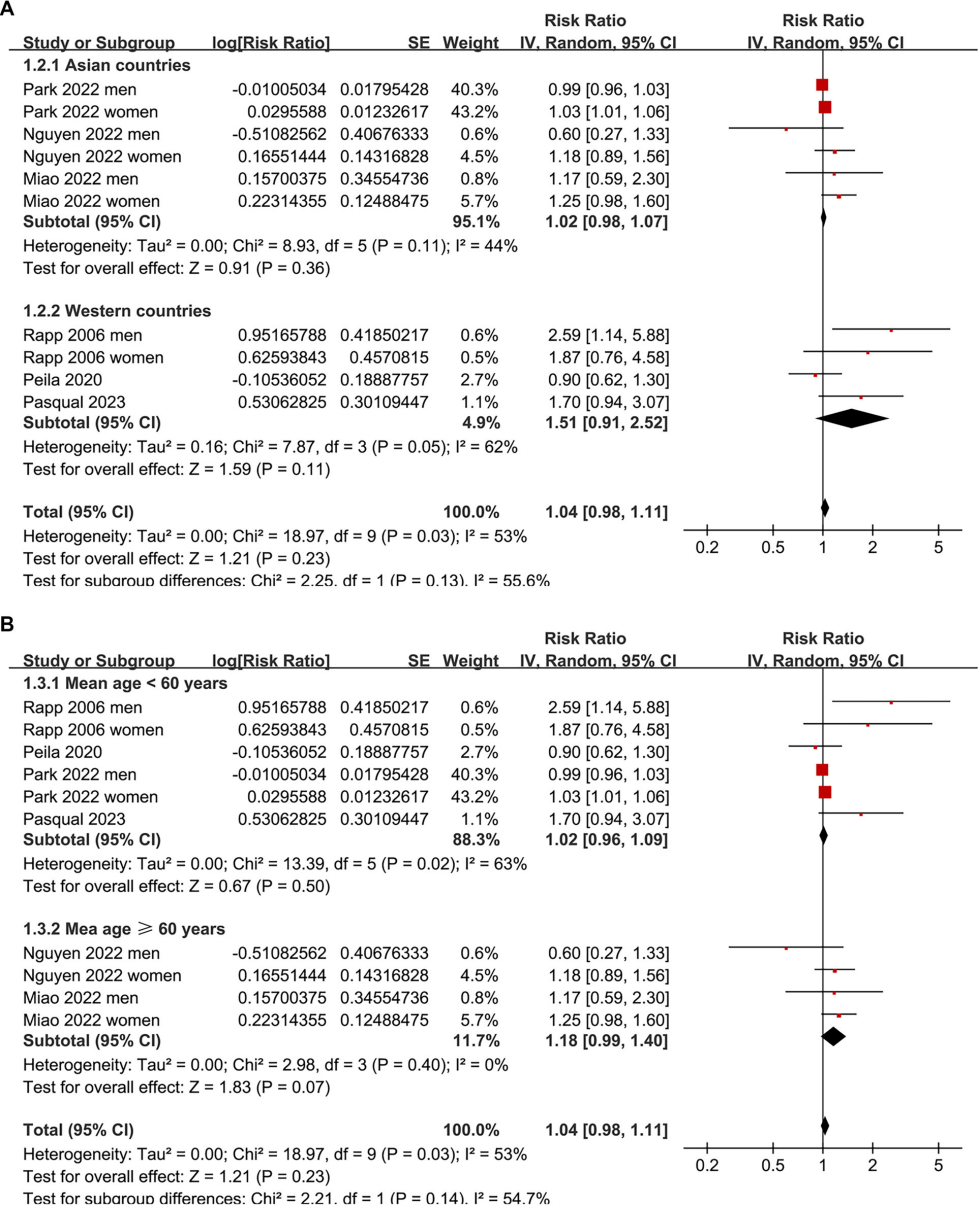
**Subgroup analyses of the association between prediabetes and thyroid cancer incidence by (A) study country and (B) mean age of participants.** Forest plots show pooled RRs and 95% CIs for thyroid cancer incidence in individuals with prediabetes compared to normoglycemia. Abbreviations: CI: Confidence interval; IV: Inverse variance; SE: Standard error; RR: Risk ratio.

**Figure 4.**
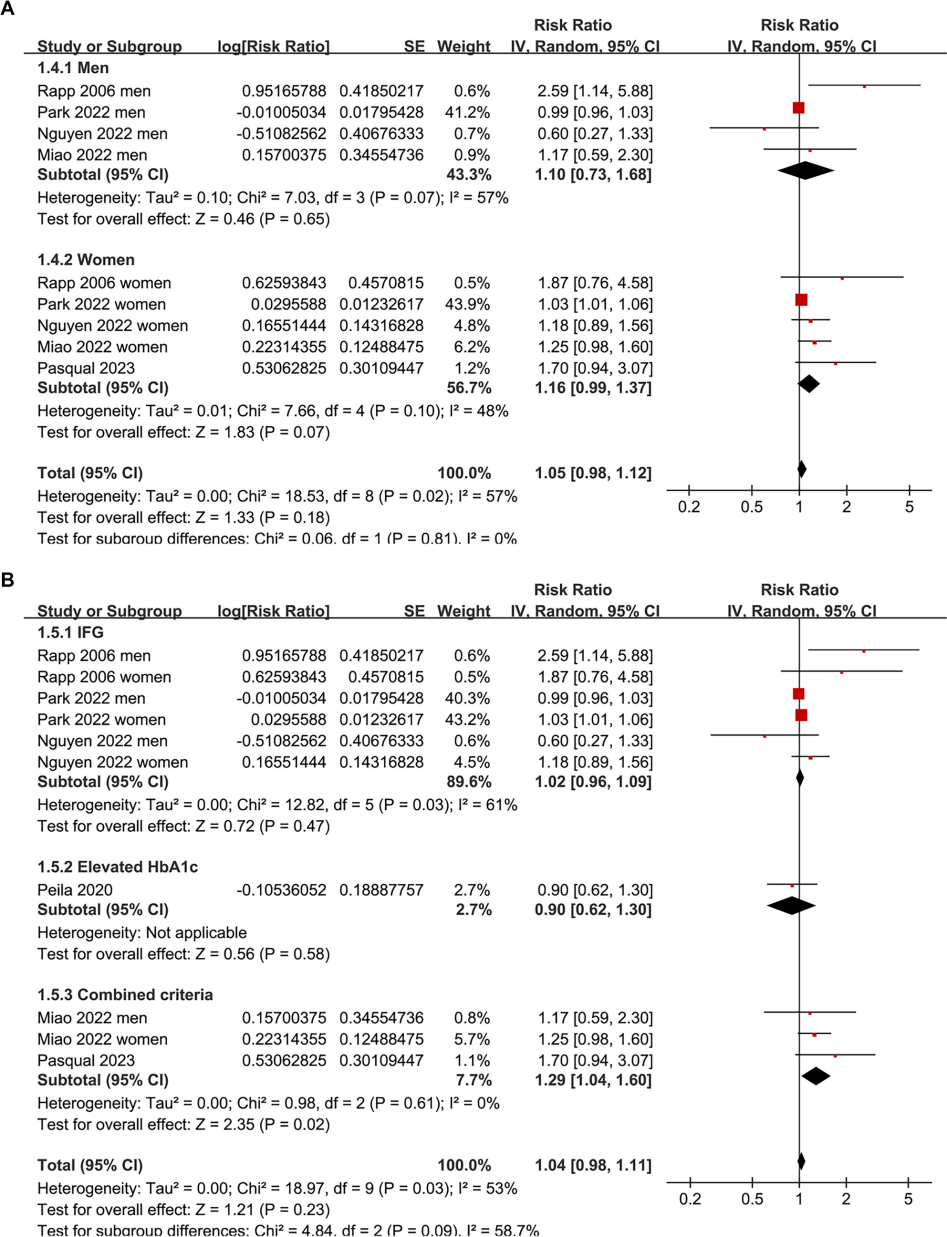
**Subgroup analyses of the association between prediabetes and thyroid cancer incidence by (A) sex of participants and (B) definitions of prediabetes.** Forest plots show pooled RRs and 95% CIs for thyroid cancer incidence in individuals with prediabetes compared to normoglycemia. Abbreviations: CI: Confidence interval; HbA1c: Glycated hemoglobin A1c; IFG: Impaired fasting glucose; IV: Inverse variance; SE: Standard error; RR: Risk ratio.

**Figure 5.**
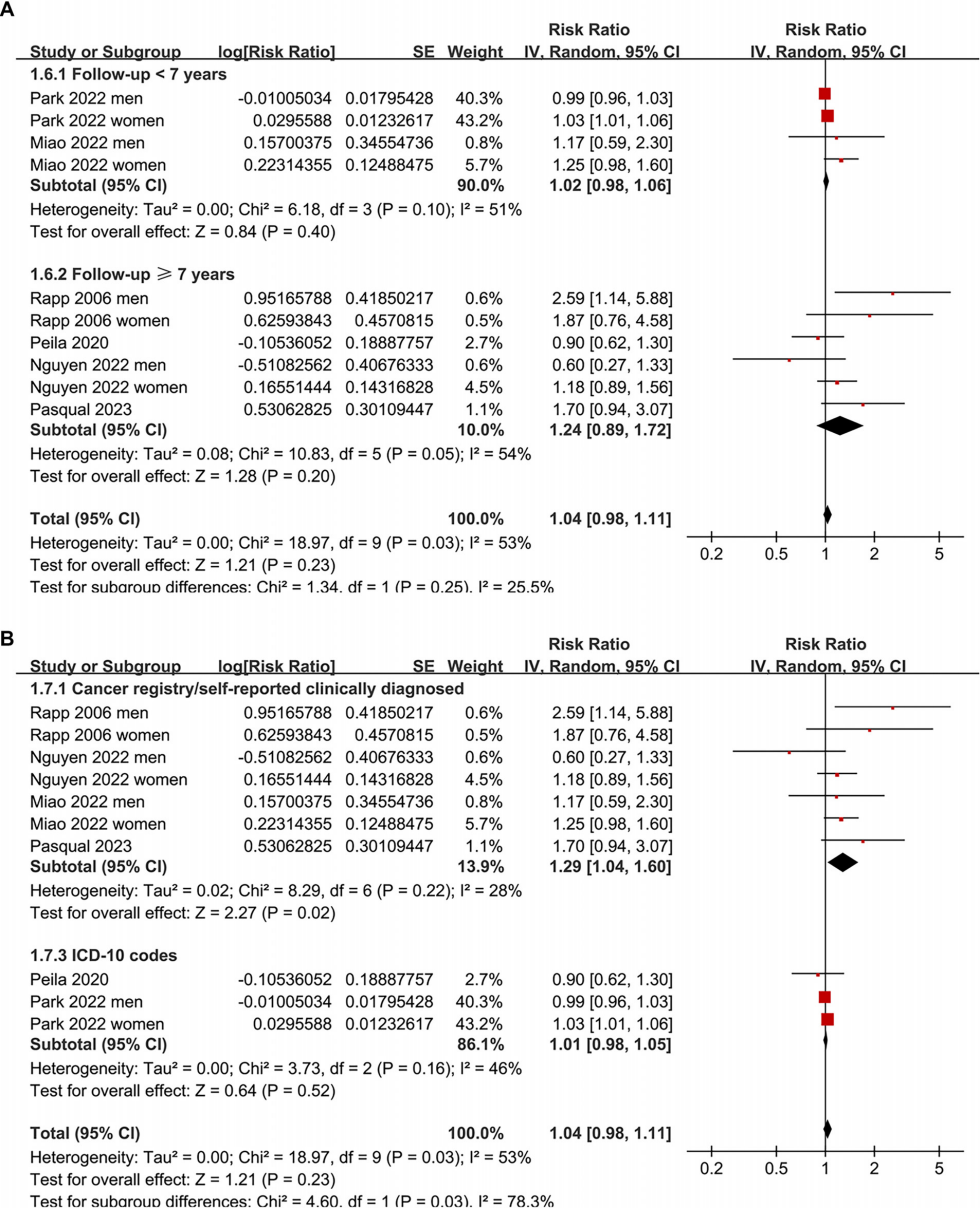
**Subgroup analyses of the association between prediabetes and thyroid cancer incidence by (A) follow-up duration and (B) method of thyroid cancer diagnosis.** Forest plots show pooled RRs and 95% CIs for thyroid cancer incidence in individuals with prediabetes compared to normoglycemia. Abbreviations: CI: Confidence interval; ICD-10: International Classification of Diseases, 10th Revision; IV: Inverse variance; SE: Standard error; RR: Risk ratio.

The pooled analysis indicated that prediabetes was not significantly associated with thyroid cancer incidence compared to normoglycemia (RR: 1.04; 95% CI: 0.98–1.11; *P* ═ 0.23; *I*^2^ ═ 53%; Figure 2). A sensitivity analysis, which excluded one dataset at a time, yielded similar results, with pooled RRs ranging from 1.03 to 1.15, all with *P* > 0.05. Subsequent subgroup analyses demonstrated that the results were not significantly influenced by study country (*P* for subgroup difference = 0.13; Figure 3A), mean ages of participants (*P* for subgroup difference = 0.13; Figure 3B), sex of participants (*P* for subgroup difference = 0.81; Figure 4A), definitions of prediabetes (*P* for subgroup difference = 0.09; Figure 4B), or follow-up durations (*P* for subgroup difference = 0.25; Figure 5A). Notably, prediabetes was associated with a significantly increased risk of thyroid cancer, as evidenced by cancer registry or self-reported clinical diagnosis (RR: 1.29, 95% CI: 1.04–1.60; *P* ═ 0.02; *I*^2^ ═ 28%). However, this association was not observed in studies using ICD-10 codes for thyroid cancer diagnosis (RR: 1.01, 95% CI: 0.98–1.05; *P* ═ 0.52; *I*^2^ ═ 46%; *P* for subgroup difference = 0.03; Figure 5B).

### Publication bias

**Figure 6.**
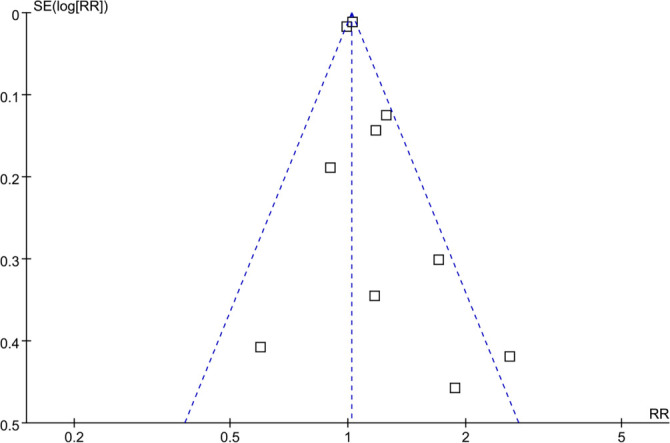
**Funnel plot assessing publication bias underlying the meta-analysis of association between prediabetes and the incidence of thyroid cancer.** The dotted line indicates the expected log[RR] under the assumption of symmetry. Abbreviations: RR: Risk ratio; SE: Standard error.

The funnel plots evaluating the association between prediabetes and thyroid cancer are illustrated in Figure 6. A visual examination of the plots indicates a symmetrical distribution, suggesting a low probability of publication bias. This finding is reinforced by Egger’s regression test, which produced a non-significant result (*P* ═ 0.58).

### Certainty of evidence

The overall certainty of evidence was rated as low according to GRADE criteria (Supplemental File 2), primarily due to inconsistencies and unexplained heterogeneity despite sensitivity and subgroup analyses.

## Discussion

In this meta-analysis of six prospective cohort studies involving over 5.7 million participants, we found no significant association between prediabetes and the incidence of thyroid cancer in the overall analysis. Subgroup analyses further indicated that this null association remained consistent across various factors, including geographic region, age group, sex, follow-up duration, and the specific criteria used to define prediabetes. These findings suggest that prediabetes, as a broad diagnostic category, may not independently confer a significantly elevated risk for thyroid cancer within the general adult population.

Notably, our subgroup analysis revealed that the method of thyroid cancer ascertainment significantly influenced the observed associations. Studies utilizing population-based cancer registries or self-reported diagnoses that were validated through medical records or pathology reports demonstrated a significantly increased risk of thyroid cancer among individuals with prediabetes (RR: 1.29; 95% CI: 1.04–1.60), with low residual heterogeneity (*I*^2^ ═ 28%). In contrast, studies that relied solely on administrative ICD-10 codes reported no significant association. This discrepancy may reflect differential misclassification; cancer registries and validated clinical records typically employ stringent diagnostic criteria and require histopathological confirmation, thereby reducing the likelihood of false positives and ensuring more accurate case identification [43]. Similarly, self-reported cancer diagnoses that are clinically validated through pathology or medical records generally exhibit high specificity [44]. Conversely, administrative data utilizing ICD codes may be susceptible to inaccuracies due to miscoding, inclusion of rule-out diagnoses, or over-diagnosis, potentially biasing the results toward the null [45].

Surveillance bias may also play a role, as individuals with prediabetes often undergo more frequent health monitoring, which increases the likelihood of cancer detection in validated settings. In contrast, such effects may be diluted in large-scale administrative databases [45]. These methodological differences highlight the critical importance of outcome validation in epidemiological research on cancer risk.

The attenuation of associations observed in these studies may therefore indicate non-differential misclassification of the outcome, potentially biasing results towards the null hypothesis.

The findings of this study align with existing literature examining the relationship between metabolic dysregulation and cancer risk. While diabetes is associated with a modest increase in the risk of thyroid cancer [46] and other malignancies [47], evidence linking prediabetes to thyroid cancer remains limited and inconsistent. Mechanistic studies suggest that insulin resistance, a hallmark of prediabetes, may facilitate tumorigenesis through hyperinsulinemia and the activation of insulin-like growth factor signaling pathways [48, 49]. Similar uncertainties regarding disease associations and the impact of outcome ascertainment have been identified in studies of non-malignant conditions. For instance, a recent meta-analysis by Jin et al. [50] found a potential link between prediabetes and Parkinson’s disease but emphasized how diagnostic methods can influence risk estimates. These parallels underscore the necessity of accurate outcome classification in research related to prediabetes.

However, it is conceivable that the degree of metabolic disturbance associated with prediabetes may be inadequate to produce a measurable effect on thyroid cancer risk, particularly in population-level analyses. Alternatively, the null association observed in most studies may reflect the influence of unmeasured or residual confounders, such as iodine intake, radiation exposure, or thyroid autoimmunity [51–53], all of which are known to affect thyroid cancer risk but were not consistently adjusted for in the studies examined. Similar observations have been noted in relation to other cancers. For example, while diabetes is linked to a higher risk of breast cancer [54], a recent meta-analysis did not find a significant association between prediabetes and an increased risk of breast cancer [55].

This meta-analysis presents several significant strengths. It represents the first comprehensive quantitative synthesis of longitudinal studies examining the association between prediabetes and the risk of thyroid cancer. The inclusion of over 5.7 million participants provides substantial statistical power to identify modest associations. All included studies employed a prospective design, minimizing the likelihood of recall bias and temporal ambiguity. Furthermore, we conducted a series of prespecified subgroup and sensitivity analyses to investigate potential sources of heterogeneity, with the risk of publication bias appearing low based on both funnel plot symmetry and Egger’s test.

However, several limitations warrant acknowledgment. First, the definition of prediabetes varied across studies, with some utilizing IFG, others employing HbA1c, and some applying combined criteria. Although this variation was addressed in subgroup analyses, it may still contribute to underlying heterogeneity. Second, the number of included studies was relatively small, and not all studies reported sex-specific or subgroup data, which limits the depth of exploration into effect modification. Third, potential confounding variables cannot be entirely ruled out, despite multivariable adjustments made in all studies. Key factors such as dietary patterns, family history of thyroid disease, and environmental exposures were not consistently accounted for.

Additionally, the observed differences in associations based on cancer diagnosis methods raise concerns regarding differential misclassification bias, which may have influenced the pooled estimates. A subgroup analysis based on the histological type of thyroid cancer could not be performed due to the absence of stratified data in the included studies. Moreover, none of the studies considered the presence of chronic autoimmune thyroiditis, a proposed risk factor for thyroid cancer [56]. The lack of data on baseline thyroid inflammation may have introduced residual confounding and limited the assessment of effect modification by underlying thyroid conditions. Finally, while Egger’s regression test did not indicate significant publication bias (*P* ═ 0.58), it is essential to recognize that this test has limited statistical power when applied to fewer than 10–15 studies. Consequently, the symmetrical appearance of the funnel plot and the negative result should be interpreted with caution.

Our findings indicate that prediabetes alone may not justify enhanced thyroid cancer screening beyond existing population-based guidelines. Nevertheless, in contexts where cancer is identified through robust and validated methods, a slight increase in thyroid cancer risk associated with prediabetes cannot be completely dismissed. These results underscore the significance of precise case identification in epidemiological research and emphasize the potential for misclassification to obscure genuine associations. Future studies should aim to utilize validated cancer outcomes and consider stratifying analyses based on underlying metabolic profiles, including insulin levels, inflammatory markers, or duration of prediabetes, to more accurately characterize at-risk subgroups. Furthermore, research involving diverse ethnic populations and varying iodine intake patterns would enhance the generalizability of these findings.

## Conclusion

In conclusion, this meta-analysis revealed no significant overall association between prediabetes and the incidence of thyroid cancer. However, a potential association was identified in studies utilizing clinically validated cancer diagnoses, indicating that the methods of outcome ascertainment may impact observed relationships. Given that all included studies were observational in nature, these findings should be interpreted with caution. Further prospective comparative studies are necessary to establish any potential causal link between prediabetes and thyroid cancer risk.

## Supplemental data

**Supplemental file 1.**

**Detailed search strategy for each database**

**PubMed**

(“Prediabetic State”[Mesh] OR “prediabetes”[tiab] OR “pre-diabetes”[tiab] OR “prediabetic”[tiab] OR “pre-diabetic”[tiab] OR “prediabetic state”[tiab] OR “borderline diabetes”[tiab] OR “impaired fasting glucose”[tiab] OR “impaired glucose tolerance”[tiab] OR “IFG”[tiab] OR “IGT”[tiab] OR “fasting glucose”[tiab] OR “HbA1c”[tiab]) AND (“Thyroid Neoplasms”[Mesh] OR “thyroid cancer”[tiab] OR “thyroid carcinoma”[tiab] OR “thyroid neoplasm”[tiab] OR “thyroid malignancy”[tiab] OR “thyroid tumor”[tiab]) Limits: Humans, English or Chinese, full-length articles Date range: Inception to April 12, 2025

**Embase**

(’prediabetic state’/exp OR ’prediabetes’:ti,ab OR ’pre-diabetes’:ti,ab OR ’prediabetic’:ti,ab OR ’pre-diabetic’:ti,ab OR ’prediabetic state’:ti,ab OR ’borderline diabetes’:ti,ab OR ’impaired fasting glucose’:ti,ab OR ’impaired glucose tolerance’:ti,ab OR IFG:ti,ab OR IGT:ti,ab OR ’fasting glucose’:ti,ab OR HbA1c:ti,ab) AND (’thyroid tumor’/exp OR ’thyroid cancer’:ti,ab OR ’thyroid carcinoma’:ti,ab OR ’thyroid neoplasm’:ti,ab OR ’thyroid malignancy’:ti,ab OR ’thyroid tumor’:ti,ab) Limits: Humans, English or Chinese, full-length articles Date range: Inception to April 12, 2025

**Web of Science**

TS=(“prediabetes” OR “pre-diabetes” OR “prediabetic” OR “pre-diabetic” OR “prediabetic state” OR “borderline diabetes” OR “impaired fasting glucose” OR “impaired glucose tolerance” OR “IFG” OR “IGT” OR “fasting glucose” OR “HbA1c”) AND TS=(“thyroid cancer” OR “thyroid neoplasm” OR “thyroid carcinoma” OR “thyroid malignancy” OR “thyroid tumor”) Date range: Inception to April 12, 2025

**Wanfang**


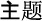
=(“
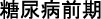
” OR “
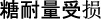
” OR “
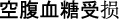
” OR “
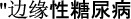
” OR “
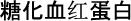
”) AND 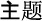
=(“
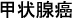
” OR “
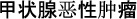
” OR “
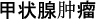
” OR “
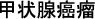
”)

English translation: Topic ═ (“prediabetes” OR “impaired glucose tolerance” OR “impaired fasting glucose” OR “borderline diabetes” OR “glycated hemoglobin”) AND Topic ═ (“thyroid cancer” OR “malignant thyroid neoplasm” OR “thyroid neoplasm” OR “thyroid carcinoma”) Limits: Human studies, Chinese language, full-length articles Date range: Inception to April 12, 2025

**CNKI**


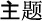
=(“
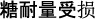
” OR “
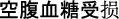
” OR “
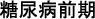
” OR “
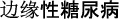
” OR “
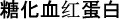
”) AND 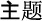
=(“
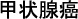
” OR “
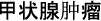
” OR “
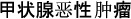
” OR “
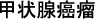
”)

English translation: Topic ═ (“impaired glucose tolerance” OR “impaired fasting glucose” OR “prediabetes” OR “borderline diabetes” OR “glycated hemoglobin”) AND Topic ═ (“thyroid cancer” OR “thyroid neoplasm” OR “malignant thyroid neoplasm” OR “thyroid carcinoma”)

Limits: Human studies, Chinese language, full-length articles Date range: Inception to April 12, 2025

**Supplemental file 2:** GRADE summary of findings

**Outcome**	**No. of Studies (datasets)**	**Study design**	**Risk of bias**	**Inconsistency**	**Indirectness**	**Imprecision**	**Publication bias**	**Overall certainty**
Prediabetes and incidence of thyroid cancer	6 (10)	Observational (cohort)	Not serious – most studies high quality; lower scores in ICD-based studies considered in subgroup analysis	Serious – moderate heterogeneity (*I*^2^ ═ 53%) only partially explained by subgroup analysis	Not serious – population, exposure, and outcome directly applicable to research question	Not serious – confidence intervals were narrow and excluded clinically large effects	None detected – symmetrical funnel plot and non-significant Egger’s test (*P* ═ 0.58)	Low

Note: The certainty of evidence was downgraded one level due to inconsistency; Despite low risk of bias and precise estimates, moderate unexplained heterogeneity in the overall analysis warranted downgrading. Subgroup analysis revealed more consistent findings in studies using validated outcomes. Abbreviations: GRADE: Grading of Recommendations, Assessment, Development, and Evaluation; ICD: International Classification of Diseases.

## Data Availability

All data generated or analyzed during this study are included in this published article.
